# Clone tag detection in distributed RFID systems

**DOI:** 10.1371/journal.pone.0193951

**Published:** 2018-03-22

**Authors:** Hazalila Kamaludin, Hairulnizam Mahdin, Jemal H. Abawajy

**Affiliations:** 1 Faculty of Computer Science and Information Technology, Universiti Tun Hussein Onn Malaysia, Parit Raja Batu Pahat, Johor, Malaysia; 2 School of Information Technology, Deakin University, Waurn Ponds, Victoria, Australia; National University of Computer and Emerging Sciences, PAKISTAN

## Abstract

Although Radio Frequency Identification (RFID) is poised to displace barcodes, security vulnerabilities pose serious challenges for global adoption of the RFID technology. Specifically, RFID tags are prone to basic cloning and counterfeiting security attacks. A successful cloning of the RFID tags in many commercial applications can lead to many serious problems such as financial losses, brand damage, safety and health of the public. With many industries such as pharmaceutical and businesses deploying RFID technology with a variety of products, it is important to tackle RFID tag cloning problem and improve the resistance of the RFID systems. To this end, we propose an approach for detecting cloned RFID tags in RFID systems with high detection accuracy and minimal overhead thus overcoming practical challenges in existing approaches. The proposed approach is based on consistency of dual hash collisions and modified count-min sketch vector. We evaluated the proposed approach through extensive experiments and compared it with existing baseline approaches in terms of execution time and detection accuracy under varying RFID tag cloning ratio. The results of the experiments show that the proposed approach outperforms the baseline approaches in cloned RFID tag detection accuracy.

## Introduction

RFID is an emerging auto-identification technology that uses radio waves to automatically identify and track physical objects without line of sight [1]. As compared to the conventional barcode, RFID tag is reusable, does not require line-of-sight, it is readable or writable and it is less error prone. As a result, RFID is expected to be a successor to the standard optical barcode and anticipated to be used in many applications including shipping and port operations [2], supply chain management [3], water level monitoring [4], anti-counterfeiting pharmaceutical products [5], banknotes [6] and also the Internet of Things (IoT) [7], [8]. As RFID enables enhanced synchronization of data as well as greater responsiveness to any changes because of real time information visibility, RFID can increase operational efficiency and lower operational cost and bring improved service quality to organizations. For example, the use of RFID technology in the supply chain management can significantly increase the accuracy as well as the efficiency and reliability of the entire chain by increasing the ability to track and locate products and distribution management. Moreover, the capability of RFID to deliver information in real-time can considerably enhances the processes of the supply chain administration and planning.

Despite its enormous advantages, security concerns have become a barrier to the widespread adoption of the RFID technology. RFID systems are vulnerable to a wide variety of malicious attacks chief of which is cloning of the genuine RFID tags [1], [9]. For example, the most widely used RFID standard Class-One Generation-Two tag [10] in critical applications such as shipping and port operations [2], supply chain management [3], pharmaceutical products [5], banknotes [6] as well as Internet of Things (IoT) [7], [8] can easily be cloned [11]. As RFID tag cloning could impose a serious threat to the RFID enabled applications and endanger the safety and health of individuals particularly in food, medical and pharmaceutical industries, these critical applications require mechanism against RFID tag cloning attacks. Furthermore, cloning of RFID tags can lead to brand damage and financial losses. The counterfeit drug market is worth of USD $40 billion per year seriously affecting the global pharmaceutical industry [12]. With RFID tags attached to drug packaging, the industry expects to substantially decrease the loss due to counterfeit drug market [12]. Without the implementation of efficient RFID tag cloning detection, the efforts to combat counterfeit pharmaceuticals will not bare fruit.

Even though RFID authentication methods that are based on cryptography and encryption are able to prevent tag cloning as well assuring privacy and security [13], these methods cannot be implemented on the low cost tags due to the resource constraint such as limited memory and computational power of the RFID tag [14]. Moreover, there is a number of well documented examples of RFID tag cloning including human implantable VeriChip tag used by Mexican government to protect access to a secure records room [15] and also Texas Instruments RFID Digital Signal Transponder (TI-DST) tag used in ExxonMobil SpeedPass systems to authenticate customers purchase gasoline [16]. TI-DST tag data is able to be captured in a short time for cracking its encryption key and this is an example that tag based security is not the ultimate solution to tag cloning. Therefore, a light weight anti-cloning approach is required to support the RFID tag clone detection.

There are several approaches for low cost counterfeit tag detection that are based on the appearance of the tags having identical unique identification (EPC) plus other related information in the system [9]–[11], [17]–[20]. However, as duplicate readings of RFID tags are common [3], [21], detection of counterfeit tag based on EPC alone cannot verify counterfeit tags from genuine tags. Advanced methods such as those that write random numbers on the tags [17], [18], [22] require redundant operations to check whether the current random number in the tag is correct and to replace it with a new random number each time the tag is read. In fact, when detection is triggered for the same EPC as in [17] and [18], manual verification is required on the objects that the tags are attached to. Certainly these approaches incur excessive overhead, large delays between the scans and slowdown the reading rate of the tags.

A recent study [20] proposed an approach using information in the e-pedigree to detect counterfeiting. However, relying on the entire certified record of the e-pedigree would not definitely verify the perfect detection of counterfeit tags. This is due to the probability of the complete e-pedigree inaccessibility in RFID-enabled supply chain [20], [23], [24]. According to [20] and [24], e-pedigree creation and management is crucial yet challenging task as its implementation involves a number of practical issues including implausibility or incompleteness. It was found that the genuine tagged products are repeatedly read with a high rate about 50.425% than the counterfeit tags [17], [18]. This is because the genuine tagged product is checked at least once at every stage of the supply chain and the counterfeit tag injected to the supply chain only after getting copied the genuine tag’s EPC which makes the scans delay.

In this paper, we propose a counterfeit RFID tag detection approach that is based on consistency of dual hash collisions and modified count-min sketch vector. The count-min sketch vector is a data structure in which we used dual independent hash functions to map the streaming tag reading data onto the sketch vector. We propose a dual verification strategy that combines consistent dual hash collisions with tag reading frequency aggregated over time intervals to verify which of the suspicious tags is genuine and which is counterfeit. Extensive performance analysis of the proposed approach is carried out and its performance is compared with baseline approaches [25]. The results of the experiments show that the proposed approach outperforms the baseline approaches as much as 99% in the detection accuracy with a reduced communication overhead under varying RFID tag cloning ratio. The contributions of this paper are summarized as follows:

- Analysis of the state-of-the-art approaches for low cost counterfeit tag detection;
- We propose a novel counterfeit RFID tag detection approach that is based on consistency of dual hash collisions and modified count-min sketch vector.
- Extensive performance analysis of the proposed approach is carried out and compared with BASE and DeClone, the approaches proposed in [25].

The rest of the paper is organized as follows: Section *Background* presents the background information and related works while Section *Clone Tag Detection Algorithm* describes in detail the proposed counterfeit tag detection approach. Performance analysis of the proposed approach is presented in Section *Performance Evaluation*. Conclusion and future work are presented in Section *Conclusion and Future Directions*.

## Background

### A. System model

**Fig 1:**
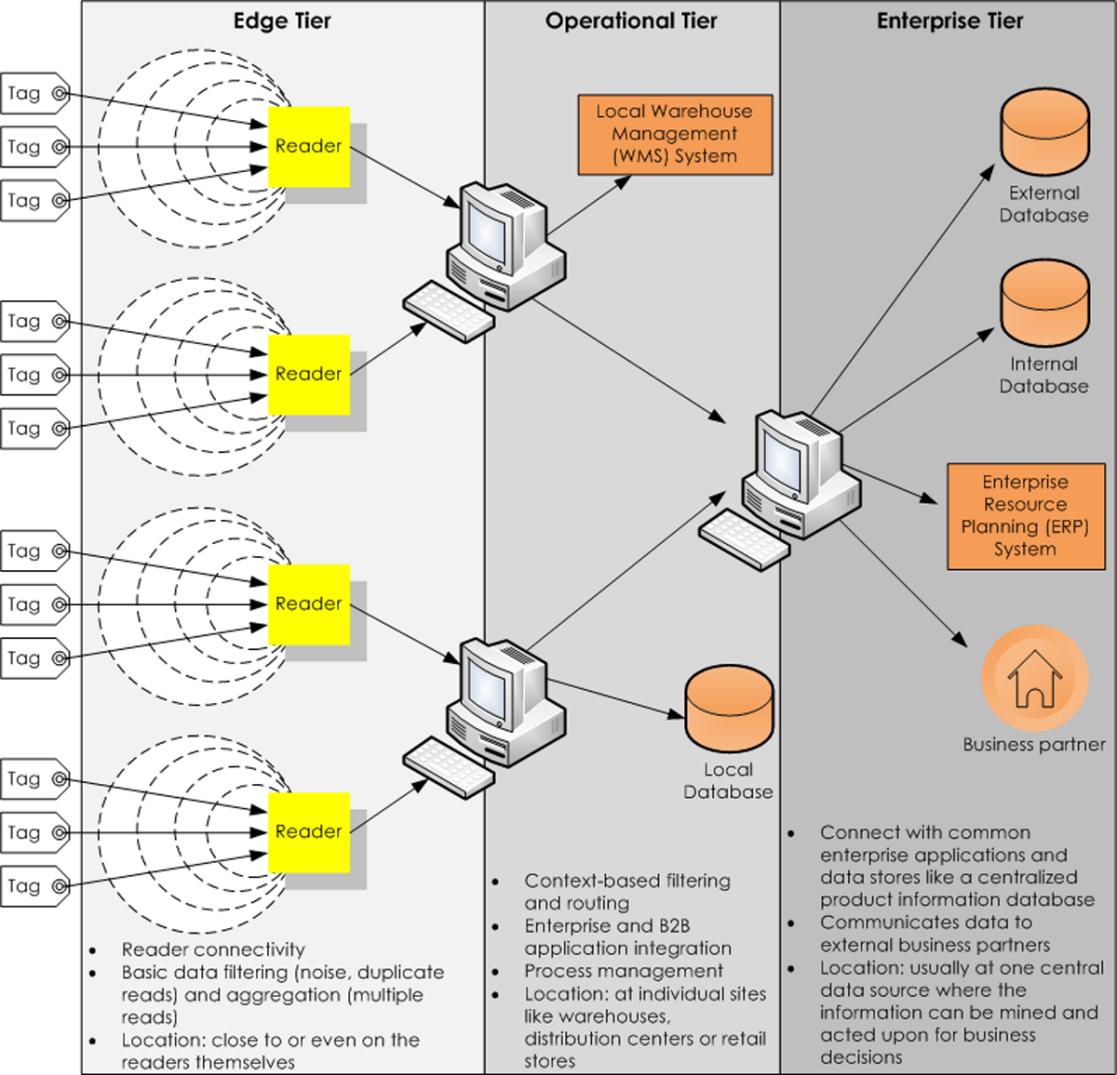
Multitier middleware architecture ([28]).

A global standard RFID data sharing infrastructure, EPCglobal network [26] is an important part of the Internet of Things (IoT). EPCglobal is made up of Electronic Product Code (EPC), EPC Information Services (EPCIS), and EPC Discovery Services (EPCDS) amongst others. Each physical product in the EPCglobal network is associated with an RFID tag, represented by a unique EPC. This EPC can be retrieved from the RFID tags wirelessly via RFID readers without line of sight. These read events are usually processed by a middleware [27], and are stored locally at each supply chain partner’s location-centric EPCIS. In order to process RFID data efficiently, middleware functionality should not be restricted to a centralized data center but rather distributed with the right level of logic placed at the right location or tier in the middleware architecture [28]. Therefore, the proposed approach (MCH) in this study is suitable to be implemented in the RFID middleware either at the operational or enterprise tier of middleware architecture for each supply chain partners (Fig 1). MCH will first do monitoring at operational tier (i.e., at individual sites like warehouse or distribution center or retail store). Overall, MCH will continually monitor EPC numbers throughout supply chain and instantly highlight any EPC numbers that are suspicious and verifying which of the tags is clone and which is genuine one.

**Fig 2:**
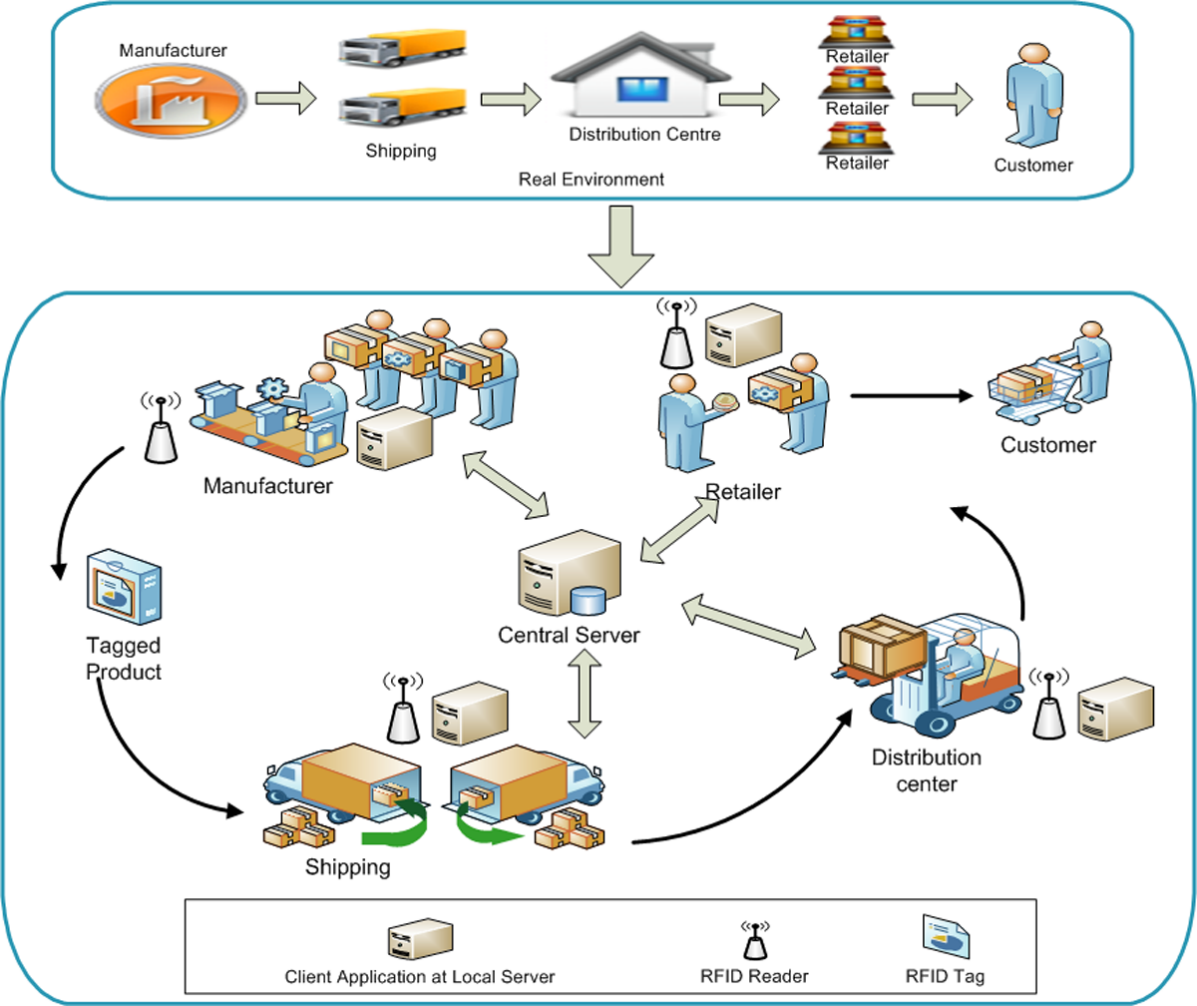
Distributed RFID supply chain system.

In this paper, we consider a distributed RFID-enabled supply chain management system as shown in Fig 2. The architecture generally consists of a backend server, RFID readers and a number of low cost passive RFID tags attached to the products. The RFID readers are interconnected with the local server via secure wired or wireless channel and communicate with the tags via wireless channel. RFID readers are placed at different locations such as at manufacturing, shipping, distribution center, retailer and checkout counters to record the product flow in distributed RFID supply chain.

Each RFID tag has a unique EPC and receives power during interrogation by a reader. An RFID reader can be any devices that capable of querying object identity stored in the RFID tag which include a PDA and a mobile phone [29], [30]. A tag interrogation by the readers is recorded in the local EPC Information Services (EPCIS). The e-pedigree data is captured via these EPCIS events and securely shared with trading partners when required [31]. For example, an RFID event could be as (EPC_02, R2, 9, t4) which elaborates that an object tagged with EPC_02 has been read 9 times by R2 (at shipping location) at time t4 for shipping. This data describes the actual path that a tagged product traveled throughout the supply chain from its start to its end which indicates transition between business phases in the distributed RFID supply chain. All the events related to a specific tag are stored in a distributed manner in the local EPCIS before synchronizing at EPCDS centralized management system to form e-pedigree [23] that can be accessed and shared by the trading partners.

Pharmaceutical industry is one of the early adopters of the passive RFID tags in their supply chain to control counterfeit medicines in the legal market [1]. However, passive RFID tags are susceptible to elementary cloning and counterfeiting attacks [1], [32]. Furthermore, since RFID readers are easily available, tracking the tag bearer is somewhat possible for the adversary to read the RFID tag and correlate its time and place to learn more about the tag. Once the tag identification is captured, the adversary can duplicate genuine tags and use the cloned tag for malicious purposes. As in [17], we assume that an adversary replicates the EPC of a counterfeit tag only when the genuine tag is manufactured and attached to product. Once the tag identification is captured, the adversary can duplicate genuine tags and use the cloned tag for malicious purposes. As RFID tags are prone to cloning, the control and monitoring of counterfeit medicines in pharmaceutical industry is a critical issue.

### B. Related work

With a wide variety of practical application of RFID tags, securing RFID infrastructure has attracted serious attention recently [33–35]. Although the problem of tag cloning has been identified as one of serious RFID security issues, it only received little attention in the literature. Presently, there are two major approaches in handling tag cloning; prevention and detection [9], [17], [18]. Prevention methods provide security against tag cloning by adopting cryptography and encryption technology to the tags. However, none of the approaches yet claim to end the cloning attack completely. Moreover, this approach cannot be implemented in the low cost tag that has been mandated for supply chain use due to constraint in the storage and computational power [14]. Therefore, detection method is the appropriate way to handle clone tag issue for low cost tags.

Several approaches concerning to clone tag detection and sketch data structure were studied. As projected in [36], events generated by clone tags are considered appear in the traces of genuine product and may cause abnormal event which can be detected as infrequent occurrence in the modeled supply chain process. In view of this scenario, example of the infrequent occurrence could be exposed by tag reading frequency of the tagged object in the modeled supply chain. This study considers an attacker replicated the EPC only when the genuine tag is ready. Therefore, the tag reading frequency of clone tag is rationally lesser than the genuine tag since time duration the clone tag exists is shorter than the genuine tag.

Even though imperfect tag reading frequency can lead to missing read or false negative, many data cleaning systems used temporal smoothing filter approach to handle this lost readings issue [37]. In that approach, a sliding window over the reader’s data stream interpolates for lost readings from each tag within the time window to provide more opportunities for each tag to be read within the smoothing window [37]. Furthermore, the experiment results presented in [17] and [18] revealed that genuine tag is repeatedly read in a high rate. Anyway, clone tags that appear before the corresponding genuine tags manufactured or after they are consumed are not considered in this study.

Through an analysis on a number of anti-counterfeiting approaches in both known and anonymous RFID systems appeared in studies between 2008 and 2016 as in Table 1, the process of clone detection for low cost tag is briefly based on appearance of tags having identical EPC. The tags with identical EPC produced tag collision known as time slot collision in Tree-based anti-collision protocols and hash collision in Aloha-based anti-collision protocols that produced the same hash digest value (output of hash function). According to [38], the use of hash values introduces possibility of tag collision among tags with the same digest. In reality, any hash function applied to different input can generate the same output due to the inherent features of the hashing. Therefore, our approach considers this by looking at consistency in dual hash collisions.

**Table 1:** Parameters for RFID tag clone detection in known and anonymous RFID systems.

**Deterministic Identification Methods** (Tag of RFID standard Class-One Generation-Two (ISO 18000-6c)			
	**Papers / Approaches**	**TID**	**Other parameters**
1	Synchronized Secrets Approach for RFID-enabled Anti-Counterfeiting [17]	Yes	The same secret random number *k*_*x*_ is stored on both the tag’s memory and the backend database.
			On every web service invocation, a new random secret *k*_*x+*1_ is generated and updated in both, the backend database and the tag’s memory.
2	Securing RFID systems by detecting tag cloning [18]	Yes	The same secret random number *k*_*x*_ is stored on both the tag’s memory and the backend database.
			On every web service invocation, a new random secret *k*_*x+*1_ is generated and updated in both, the backend database and the tag’s memory.
3	Fast cloned-tag identification protocols for large-scale RFID systems [19]	Yes	Establish expected reading list and compare with actual reading list
4	Exposing Clone RFID Tags at the Reader [11]	Yes	Clone tags are trivially evident on the basis that multiple EPC’s of the same value were obtained in a single inventory cycle (clones need to appear in the same tag group, and at the same reader in time)
5	DTD [10], [40]	Yes	1^st^ track—Verification information is written on tag as products flow along the supply chain which forming verification sequences
			2^nd^ track—Check on consistency of business transaction performed during the supply chains
			The verification sequence together with the sequence formed by business actions performed during the supply chains yield two tracks which can be assessed to detect the presence of clone tags
6	TDPS [20]	Yes	Product e-pedigrees in manufacturing to facilitate RFID-based track-and-trace anti-counterfeiting.
7	Tailing RFID Tags for Clone Detection [9]	Yes	RFID readers write random values to tags as they pass through a supply chain, creating in each tag a tail composed of random values.
			The tails of legitimate tags and clone ones diverge over time, making cloning detectable by a centralized detector even across blind zones.
**Anonymous RFID systems** (Tag of RFID standard Class-One Generation-Two (ISO 18000-6c) Detecting anonymous clones requires solutions that accept tag IDs as “black boxed”			
	**Papers / Approaches**	*TID	**Other parameters**
1	GREAT [39]	Irreconcilable collisions	Using Aloha-based anti-collision protocol to find irreconcilable collisions.
			GREAT used slotted Aloha *h*(*f*,*r*,*ID*) to find possible irreconcilable collisions.
2	BASE [25]	Irreconcilable collisions	ID cardinality and tag cardinality.
			BASE used slotted Aloha *h*(*f*,*r*,*ID*) to find possible irreconcilable collisions.
3	DeClone [25]	Irreconcilable collisions	Uses a hybrid design of slotted Aloha and tree traversal (Breadth First tree traversal-BFS) to determine collisions.
			DeClone used slotted Aloha *h*(*f*,*r*,*ID*) to find possible irreconcilable collisions.
4	DCTD [22]	Irreconcilable collisions	Using a Tree-based anti-collision algorithm to find irreconcilable collisions by dividing the tags that answer the query in collision time slots into many different groups until each group have only one ID.
			Tags with the same ID are always divided into the same group, and then gives rise to an irreconcilable collision.
			Adopt the Manchester code to speed up finding out irreconcilable collisions.
			Each tag is preloaded with a unique secret pseudonym. After a successful authentication between a tag and the legal reader, the pseudonym stored both in the backend server and in the tag should be updated privately.
			The reader sends a query prefix at first, and then the tags in the reader’s work range respond the query only if their own ID contains this prefix.

Clone detection through identical EPC not only applicable in known RFID system but also in anonymous RFID system as studies in [39] and [25]. GREAT [39], BASE [25] and DeClone [25] are clone tag detection approaches in anonymous RFID system and used slotted Aloha to find any hash collision that caused possible irreconcilable collision due to identical EPC. GREAT is an approach that is based on framed slotted Aloha anti-collision and detects the clone tag probabilistically while DeClone is the improved approach of similar groundwork with addition on the Breadth First tree traversal (BFS).

Fast clone tag identification protocols for large-scale RFID systems [19] required more spaces to store the expected and actual reading list while comparison between the lists gives significant impact on the execution time. GREAT [39] adopts probabilistic arbitration protocol and therefore only tolerates a few clones. Besides, execution time of GREAT tends to be infinite if used to detect 100% clone tags. In BASE [25], the amount of tag and amount of EPC is compared for the reason that clone attack makes tag quantity exceed the EPC quantity. However, this approach is less efficient for large scale system because clone tags might respond at the very beginning of the protocol execution yet BASE needs to count almost all tags until it detects the tag quantity exceed the EPC quantity. Table 2 provides the summary of RFID clone tag detection approaches.

**Table 2:** Summary of RFID clone tag detection approaches.

	Approaches	Weaknesses
1	DTD [10], [40]	The rules indicated still rely on a predefined structure of supply chain (business transaction) and therefore it is not flexible for dynamically change supply chain as the author claimed.
		Great reliance on product movement information from e-pedigree.
2	Fast cloned-tag identification protocols for large-scale RFID systems [19]	The approach involves establishing expected reading list and compare with actual reading list, thus it required more spaces to store the expected and actual reading list while comparison between the lists gives significant impact on the execution time especially for large scale systems.
3	GREAT [39]	Cannot detect all clone tags completely and the detection performance is probabilistic because of bounded-ness of the frame slotted Aloha anti-collision adopted.
		Find out irreconcilable collisions in a probabilistic way therefore tolerate only a few clones.
		Execution time of GREAT tends to be infinite if used to detect 100% clone tags.
4	Securing RFID systems by detecting tag cloning [18]	Used two parameters, similar EPC and secret random number on every tag to detect clone tag in which unsynchronized secrets are another proof of a tag cloning attack.
		However the presented method still needs to be used together with a manual inspection to determine which of the objects is clone under different cases.
5	BASE [25]	Tag and EPC quantity is compared because a cloning attack makes tag quantity exceed EPC quantity. BASE needs to count almost all tags until it detects the cloning attack.
		Thus, it is less efficient for large scale (more than 1000 tags) because clone tags might respond at the very beginning of the protocol execution.
6	DeClone [25]	Even though it claims that clone tag can be detected when at least one of the slots allocated get only one EPC hashed into, it still uncertain to differentiate which of the suspicious tag is clone and which is genuine.
7	DCTD [22]	It still uncertain to differentiate which of the suspicious tag is clone and which is genuine.

Another anti-counterfeiting approach in anonymous RFID systems, DCTD [22] was developed based on Tree-based anti-collision protocols. A pseudonym method is chosen to prevent possible leakage of tag IDs in the detection process, and the Manchester code is adapted to speed-up finding irreconcilable collisions. DCTD preloaded each tag and backend server with unique secret pseudonym and updated privately after every successful authentication between tag and the legal reader. When reader sends a query prefix, the tag responds the query only if it’s pseudonym contains this prefix. The approaches in [19], [22], [25], [39] reveal that cloning of the genuine EPC can be triggered by tag collision not only in known RFID systems but also in anonymous RFID systems. However, the approaches required genuine and clone tags to be presented at the same time and location.

Studies in [17] and [18] allocated a unique EPC and a secret random number on every tag as well as study in [22]. A record of tag EPC and its corresponding secret random numbers are stored and synchronously changed in both the tag and backend database server. Random number on tag’s memory will be rewrite and updates when reader reads the tag. Clone tag is detected when reader reads tag with different random number as stored in the backend server. Study in [9] apply almost similar approach for clone tag detection as in [17] and [18]. In [9], the reader writes random number to tag as it pass through supply chain and constitute a tail. The tails of genuine tags and clone ones are inconsistent over time and therefore making the clone tag be identified by comparing these tails. BASE [25], DeClone [25] and DCTD [22] for instance aim for anonymous RFID system which EPC is unknown. Eventually these approaches rely on tag collision due to identical EPC to detect the clone existence. Therefore this baseline is adapted to our approach which is focus on known RFID system.

### C. Sketch vector data structure

In line with [41], storing streaming data in memory can be done efficiently using sketch. According to [42], a sketch is a summary data structure that requires storage which is significantly smaller than the input stream length. Sketch based methods liked count-min sketch [42] is using hashing to map items in the streaming data onto a small-space sketch vector that can easily be updated and queried. The count-min sketch modeled the data stream as a vector *a*(1‥*K*) and use *d* pairwise independent hash functions {*h*_1_‥*h*_*d*_}. Pairwise independence is a method to construct a universal hash family, a technique that ensures lower number of collisions in the hash implementation.

Recently, sketch techniques have been used in frequent item mining [43], [44] and anomaly detection [45]. According to [46], sketch techniques can be used to perform distributed computation of aggregates without the need to send the actual data values. The tight connection with both data streaming and distributed computation makes sketching techniques important from both the theoretical and practical point of view. Approach in [43] used sequential sketch approach to create hash-compressed representations before mining frequent sequential patterns of uncertain time series data stream.

Approach in this study apply a modified count-min sketch with two independent hash functions in observing identical EPC in local site and distributed region in supply chain. The appearance of identical EPC can be endorsed through consistency of dual hash collisions in the modified count-min sketch vector data structure. We consider the tag reading count and time are constantly updated in the same sketch of each reader. When certain point of time is met, record of the tag readings can be removed from the sketch. Clone tags that appear before the corresponding genuine products or tags are manufactured or after they are consumed are not considered in this study.

## Clone tag detection algorithm

In this section, we described how the proposed approach detects and verifies the presence of a cloned tag in distributed RFID system with sketch vector. The algorithm is design for controlled environment where there is time boundary for each tag to arrive at each location. This setting is a norm in manufacturing fields where objects moves by their schedule.

### A. The proposed approach

**Fig 3:**
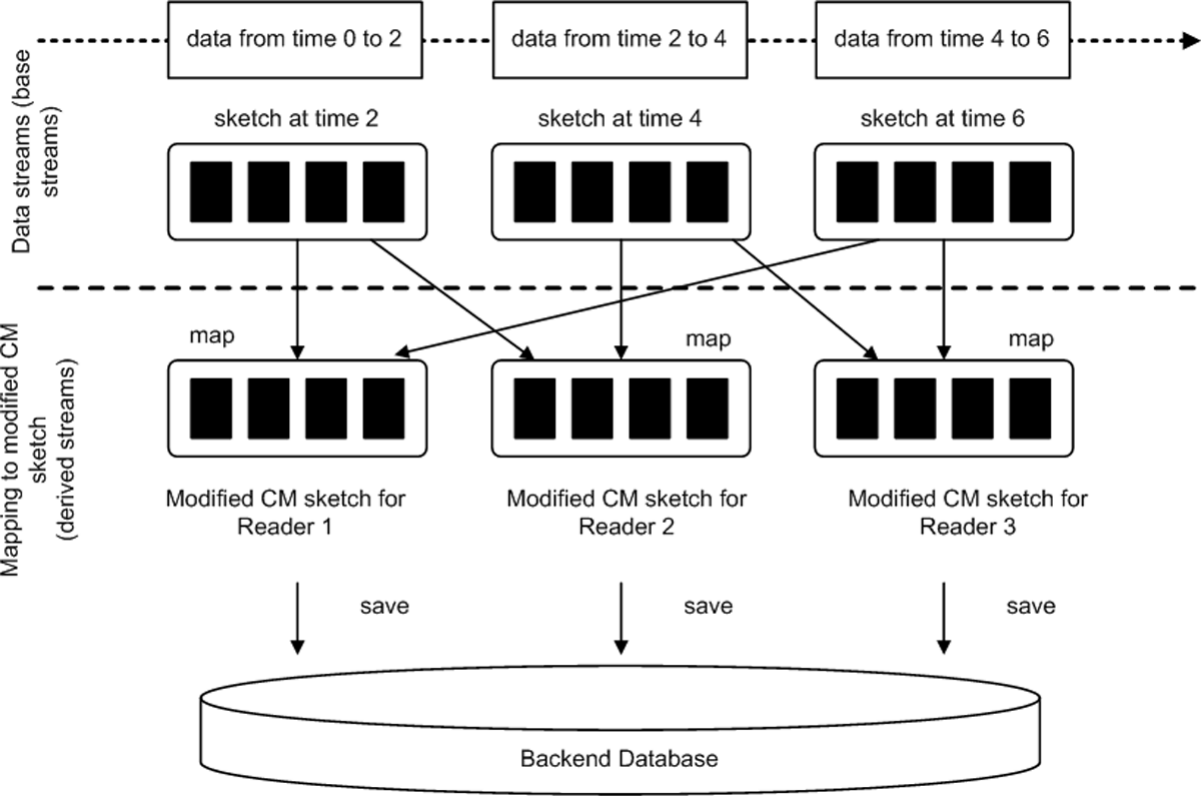
Mapping of base stream into modified count-min sketch.

We refer to Fig 3 for the description. We assume that the RFID tag readings are in a form of data stream [21]. Let *S* = {*sketch*_1_, *sketch*_2_,…, *sketch*_*M*_} denotes a data stream of tag readings that is divided into batches of *T* seconds where *M* ≥ 1. Since the data stream is unbounded stream, it is divided into batches of *T* times (example within a number of epochs) (e.g. 2 epochs * 2.5s per epoch = 5s) for processing. Internally, the data stream is a sequence of sketches, one for each batch interval (e.g. batch data in 5s).

The sketch is a distributed collection of tag readings that is spread out across multiple RFID readers collaborated between the supply chain partners. Each sketch contains the tag reading records received during the batch interval. Data contained in the sketches are partitioned into a set of modified Count-Min (*CM*) sketches. Let *CM =* {*CM*_1_, *CM*_2_,*…*, *CM*_*N*_} denotes a set of modified *CM* sketches where *N* > 1 Data in the sketches are partitioned and cached in memory of central server based on RFID readers involved (the message values are parsing into Reader objects). Let *R* = {*R*_1_, *R*_2_,…, *R*_*N*_} denotes the set of RFID readers involved in the supply chain. Assume there is just one base stream containing tag reading information with schema:

ReadingInfo(tagID,reader,readcount,time)

Attribute *tagID* identifies the tag EPC, *reader* denotes the reader that read the tag (also represent location where the tag is read), *readcount* denotes the number of read occurrence and *time* denotes the tag reading time. Following is example of query to map data contained in the sketches into modified *CM* sketch of specific reader:

FOR *CM*_*N*_

SELECT *

FROM *S*

WHERE *sketch*.*reader = = N*

Let *h*_1_ and *h*_2_ represent the hash function for the first row and the second row of each *CM*. Let *R*_*c*_ *=* {*R*_*c*1_, *R*_*c*2_} ⊂ *R*, where *R*_*c*1_ denotes a set of readers that are involved in hash collision using hash function *h*_1_ and *R*_*c*2_ denotes a set of readers that are involved in hash collision using hash function *h*_2_ The following are examples of query to find *R*_*c*1_ and *R*_*c*2_ (TRUE if hash collision occur):

SELECT *R*_*c*1_

FROM *R*

WHERE *h*_1_ (*R*_1_.*tagID*,*…*,*R*_*N*_.*tagID*) = TRUE

SELECT *R*_*c*2_

FROM *R*

WHERE *h*_2_(*R*_1_.*tagID*,*…*,*R*_*N*_.*tagID*) = TRUE

Let *R*_*f*_ = *{R*_*f*1_,*…*,*R*_*fx*_*}*⊂*R*_*c*_ denotes set of readers where *X*>1 that ultimately having hash collision at both hash functions *h*_1_ and *h*_2_ if and any if the *tagID* is equal. Let *EqualTagID* represent function to check if the *tagID* is identical (TRUE if *tagID* is identical). Following is example of query to find *R*_*f*_:

SELECT *R*_*f*_

FROM *R*_*c*1_, *R*_*c*2_

WHERE *EqualTagID(R*_c1_.*tagID*, *R*_c2_.*tagID)* = TRUE

For an identical *tagID*, *R*_*f*_.*readcount* updated in the *CM*s are compared. We consider the genuine tag is the one that having greater *readcount*, otherwise the tag is clone. To demonstrate the proposed approach, we have already implemented it in a specific case study as demonstrated in the following section.

### B. RFID data stream

Readers interrogate adjacent tags by sending out radio frequency (RF) signal. RFID tags in the area respond to these signals with their unique EPC. Technically a tag can be read one at a time in very rapid succession. The process happens very quickly such that it seems like the reader is interrogating many tags at once. However, for a very dense tag population, the tags would need to be in the read field for few seconds [47]. According to [48], when a reader sends a signal to determine all tags in its reading vicinity, it is known as single interrogation cycle. The results from a number of interrogation cycles are grouped into an epoch that is specified as a unit of time which typically ranges between 0.2–0.25 seconds. Within this time, the reader keeps track of all the tags it has identified, as well as additional information such as the number of interrogation responses for each tag and the time at which the tag was last read. The information is stored internally in a tag list which is periodically transferred to the reader’s client [48]. The approach in this study receives the reading list by all the connected readers periodically for mapping, updating and cloning checks.

In line with [48], this study mapped RFID readings statistically. The observed readings can be viewed as a random sample of tags population in the physical world. The number of tag reading frequency in an epoch is a random variable that follows Binomial distribution as work in [48]. The observed reading frequency for such tag during an epoch is sampled in conjunction with the known number of interrogation cycles per epoch. As depicted in Table 3, by assuming a reader configured with a total number of 10 interrogation cycles per epoch and the overall tag reading frequency in the major detection region is around 80%, the reading frequency differ across tags and can vary over times as the observed tags move within the reader’s detection range. The reading frequencies stored in the tag lists submitted by the readers are employed as input to the proposed approach. The updated tag reading frequency is preserved as second parameter for clone check.

**Table 3:** Tag reading data.

Reader 1			Reader 2			Reader 3		
EPC	Read Count	Time	EPC	Read Count	Time	EPC	Read Count	Time
1	10	t1	7	10	t2	6	7	t3
2	7	t1	8	10	t2	12	9	t1
3	9	t1	5	2	t4	17	8	t2
4	10	t1	6	1	t4	18	10	t1
2	7	t2	2	3	t4	5	8	t2
6	8	t2						
2	7	t3						
7	2	t3						
8	3	t3						
5	9	t3						

### C. Mapping tag reading to modified count-min sketch

According to [49], sketch property are perfectly suitable for both data streaming and distributed computation, since they can be updated on pieces. With some modifications, this study implemented the count-min (*CM*) sketch data structure introduced by [42]. The *CM* sketch modeled the data stream as a vector *a*(1‥*K*) and use *d* pairwise independent hash functions {*h*_1_‥*h*_*d*_}. Pairwise independence is sometimes called as strong universality. Each of the hash function hashes each of the input (EPC) into uniformly random integer in the range (*1*‥*K*) where *K* is the quantity of home buckets. The data structure itself consists of two dimensional array with size (space used) K*h cells with length of *K* and width of *h*. Each hash function matches to one 1-dimensional array with *K* cells.

When an update (*i*_*t*_,*c*_*t*_) comes from the stream, hash functions are used to determine the counter position for updating the sketch by hashing the *i*_*t*_ and add the *c*_*t*_ to the corresponding cell in each row. Linked nodes and home buckets are applied in the original count-min sketch to reduce a one-to-one correspondence between record addresses and possible tags read. Furthermore, this technique is to minimize slot collision issue which will strictly eliminate insertion of new tag reads. Sketch vector applied in this study includes two dimensional array denoted by *CM*[*d*,*K*]. *d* is the number of hash functions *h*(*d*) and *K* is the quantity of home bucket which is also the maximum hash value range (uniformly random). For example, let *h*_*j*_ be the *j*-th hash function in *h*(*d*):*CM*[*0*,*…*,*k*] that hash the EPC for record address and store its EPC (*tagID*), location (*reader*), reading count (*readcount*) and reading time (*time*) to the *j*-th row at the *h*_*j*_*(EPC)* column. Initial value for each element in the *CM[d*,*k]* is set to 0. For repeated tag read, the attributes update for example reading count is added to *CM*[*j*,*h*_*j*_(*EPC*)] as in Eq 1.

$$CM\left[j,h_{j}\left(EPC\right)\right]=CM\left[j,h_{j}\left(EPC\right)\right]+readcount$$ (1)

**Fig 4:**
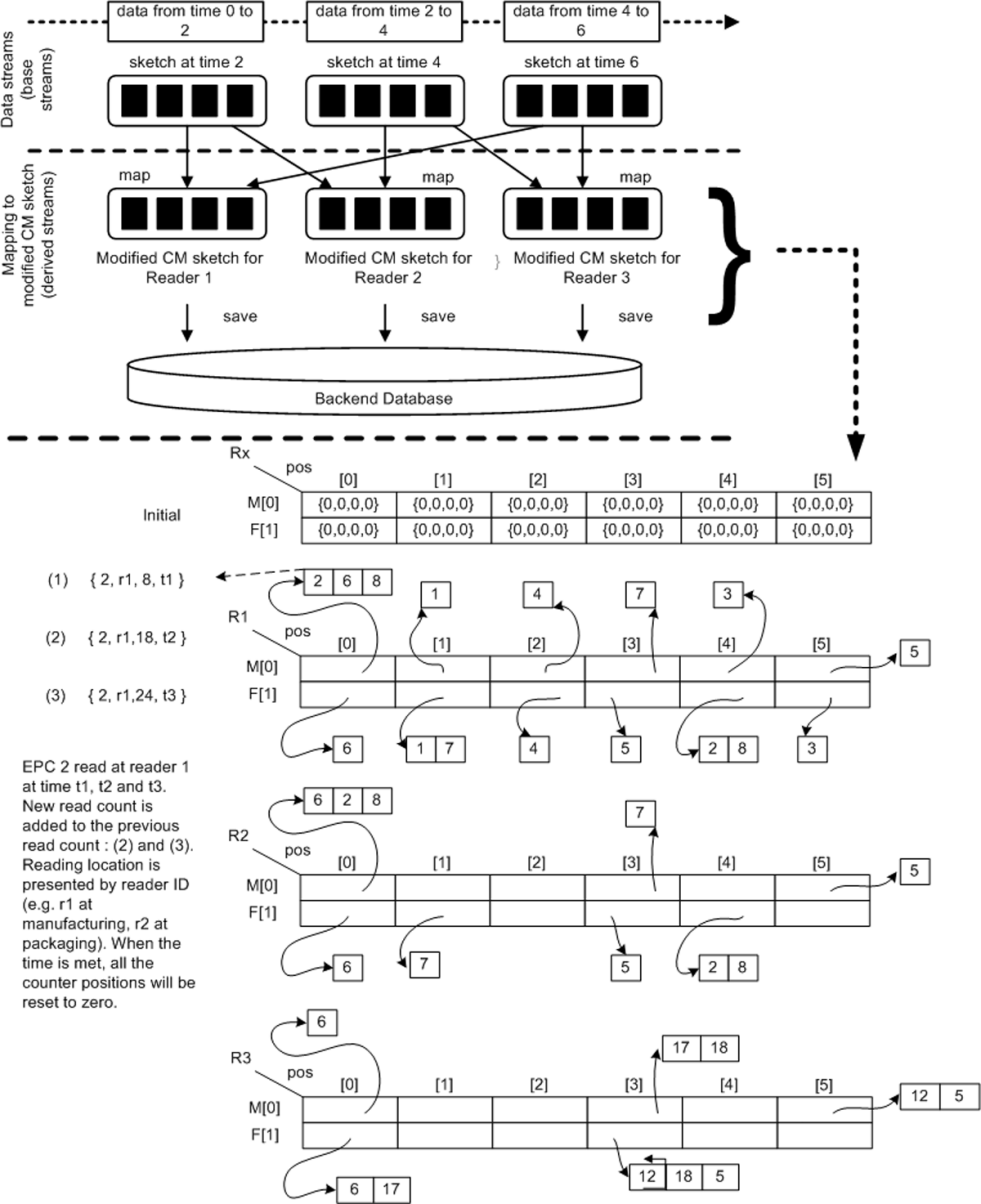
*CM* sketch visualization of initial, map and update reading for three readers.

The following illustration Fig 4 demonstrated the proposed approach based on sample data of tag reading in Table 3. Fig 4 illustrates the *CM* sketch visualization of mapping and update reading for three readers. At initial point, all counters are set to 0. Each EPC is mapped to one slot in each row of the particular *CM* sketch. For every slot address resulted from both hash functions used, a bucket is created that will contain an item or linked items in the case of collision at the same slot.

Table 4 shows the content of each *CM* sketch vector used for every reader in Fig 4. The table prints out content of all non-empty buckets and its item or corresponding linked items.

**Table 4:** Print vector content.

Reader 1	Reader 2	Reader 3
2 → 6 → 8 →		
1 →	6 → 2 → 8 →	6 →
4 →	7 →	17 → 18 →
7 →	5 →	12 → 5 →
3 →	6 →	6 → 17 →
5 →	7 →	12 → 18 → 5 →
6 →	5 →	
1 → 7 →	2 → 8 →	
4 →		
5 →		
2 → 8 →		
3 →		

### D. Managing counterfeit hash algorithm

We now explain the proposed clone tag detection algorithm which we refer to as the Managing Counterfeit Hash (MCH). The proposed approach considers two different but interrelated steps for evaluating the presence of clone tags: (i) consistency of dual hash collision at different *CM* sketch vectors (different readers) and (ii) the tag reading frequency.

A cloned tag has a duplicate copy of EPC of a genuine tag. When a reader reads the tags, it cannot differentiate between the two tags. However, when hashing the same EPC using the same hash function, hash collisions occur because the hashing process produced the same hash digest value. Hash collision produced from the hash function represents slot collision in the *CM* sketch vectors. Our approach relies on consistency of hash collision by two hash functions in the different *CM* sketch vectors to reveal the presence of clones. As noted earlier, an adversary creates cloned tags after the genuine tag is ready, the tag reading frequency of the cloned tag is reasonably lesser than the genuine tag. Thus, if a hash collision occurs and there exists identical EPC at both *CM* sketch vectors, constantly updated reading frequency may determine precisely at which reader the clone tag exists.

Algorithm 1 shows the pseudo code of the proposed clone tag detection algorithm in distributed RFID system. The core input to MCH is sequence of sketches that consist tag reading information with attributes *tagID*,*reader*,*readcount* and *time*. At lines 1–3, MCH will first check the time to remove all the readings. If the time is met, all the counters will be reset to zero. Next, at line 5, reading from each reader is sent in form of base data stream to the central coordinator which will execute this approach. For mapping and update at line 5–20, each incoming reading from sketches will be mapped to *CM*_*N*_ if the reader is *N*. In the specific *CM* sketch, the *tagID* will be hashed using two hash functions and the output is considered as the counter position into the sketch vector (line 8–9). At line 10–13, the algorithm first checks to see if counter position for the hash digest value is filled at particular sketch vector. If null, bucket is created and the read tag item is added to the bucket. If not null and if the item count not exceeds bucket size, the read tag item is added to the bucket tail. If item count exceeds the bucket size, the bucket is considered overflow. For clone detection at line 21–32, if equal *tagID* traced in the similar bucket position in at least two readers at once, *readcount* of the *tagID* is compared (line 27). *tagID* that has less *readcount* is considered as clone tag. Therefore, this can verify at which reader the clone tag exists precisely.

Algorithm 1: MCH

Input: sketch

BEGIN

1. **IF** (Time = True) **THEN** //empty the space
2.     *CM*_*N*_={0}
3. ENDIF
4. //map and update
5. **WHILE** reading from sketch
6.     //map to *CM*_*N*_ IF *sketch*.*reader*=*N*
7.     For each *CM*_*N*_ do:
8.       **FOR (i = 1 TO d)** //insert data at *h*_*i*_
9.         *position*_*i*_←h_j_(tagID)
10.           **IF** (*CM*_*N*_[h_j_, *position*_i_]=*NULL*) **THEN**
11.             //create bucket and add item
12.           **ELSE IF** (*CM*_*N*_[*h*_j_, *position*_i_]≠*NULL*) **THEN**
13.             //add linked item
14.           **END IF**
15.           **IF** (*sketch*.*tagID*=*CM*_*N*_.*tagID*) **THEN**
16.             //update *readcount* and *time*
17.           **ELSE** //add item to the bucket tail
18.           **END IF**
19.         **END FOR**
20. **END** **WHILE**
21. // clone detection
22. Find *R*_*c1*_ and *R*_*c2*_ from *R*
23. Find set of *R*_*f*_ from *R*_*c1*_ and *R*_*c2*_
24.           **IF**(*R*_*c1*_.*tagID*=*R*_*c2*_.*tagID*) **THEN**
25.              **RETURN** *R*_*f*_
26.              For each *R*_*f*_ do:
27.                 **IF** (*R*_*f*_.*readcount* is greater) **THEN**
28.                 // tag at *R*_*f*_ is genuine
29.                 ELSE // trigger alarm to indicate clone tag detected at *R*_*f*_
30.           **ELSE**
31.             // genuine tag
32.         **END IF**

END MCH

                Algorithm 1: MCH

Referring to the example illustrated in the previous section, we executed experiment that identifies clone tag at three different readers by assuming the readers are distributed at different places. We apply principle that genuine tag is read in a high rate. Clone tag detection can be identified on at least two readers at once in order to accurately trigger existing of clone and at which reader. Furthermore, the similar clone EPC may not exist at all readers. If considering all readers in *R*_*f*_ that having identical EPC simultaneously, the genuine tag is considered having higher reading frequency.

Table 5 shows the results example of our clone detection approach. The results point out that clone is correctly measure if it is traced twice (1^st^ and 2^nd^ trace) on the same tag and both reporting the clone tag exist at similar reader (e.g. 1^st^ trace at reader 2 (r2) and 2^nd^ trace also at reader 2 (r2)). The 1^st^ and 2^nd^ trace represents that the similar EPC triggered slot collision at both hash functions. Table 6 illustrates the position of tag reading in particular *CM* sketch with updated reading rate.

**Table 5:** Clone check results at three readers.

**Results of Clone Check between R1 and R2**
2 → 1^st^ trace of clone at r2
2 → 2^nd^ trace of clone at r2
5 → 1^st^ trace of clone at r2
5 → 2^nd^ trace of clone at r2
6 → 1^st^ trace of clone at r2
6 → 2^nd^ trace of clone at r2
7 → 1^st^ trace of clone at r1
7 → 2^nd^ trace of clone at r1
8 → 1^st^ trace of clone at r1
8 → 2^nd^ trace of clone at r1
**Results of Clone Check between R1 and R3**
5 → 1^st^ trace of clone at r3
5 → 2^nd^ trace of clone at r3
6 → 1^st^ trace of clone at r3
6 → 2^nd^ trace of clone at r3
**Results of Clone Check between R2 and R3**
5 → 1^st^ trace of clone at r2
5 → 2^nd^ trace of clone at r2
6 → 1^st^ trace of clone at r2
6 → 2^nd^ trace of clone at r2

**Table 6:** Tag reading in particular *CM* sketch with updated reading rate.

Reader 1	Reader 2	Reader 3
r1[0][222] → 8(read: 3) r1[0][598] → 5(read: 9) r1[0][805] → 2(read: 21) r1[0][885] → 4(read: 10) r1[0][968] → 3(read: 9) r1[0][1061] → 6(read: 8) r1[0][1227] → 7(read: 2) r1[0][1463] → 1(read: 10)	r2[0][222] → 8(read: 10) r2[0][598] → 5(read: 2) r2[0][805] → 2(read: 3) r2[0][1061] → 6(read: 1) r2[0][1227] → 7(read: 10)	r3[0][598] → 5(read: 8) r3[0][920] → 18(read: 10) r3[0][1061] → 6(read: 7) r3[0][1277] → 17(read: 8) r3[0][1637] → 12(read: 9)
r1[1][48] → 5(read: 9) r1[1][261] → 4(read: 10) r1[1][615] → 3(read: 9) r1[1][794] → 8(read: 3) r1[1][828] → 2(read: 21) r1[1][1148] → 7(read: 2) r1[1][1182] → 1(read: 10) r1[1][1361] → 6(read: 8)	r2[1][48] → 5(read: 2) r2[1][794] → 8(read: 10) r2[1][828] → 2(read: 3) r2[1][1148] → 7(read: 10) r2[1][1361] → 6(read: 1)	r3[1][48] → 5(read: 8) r3[1][893] → 17(read: 8) r3[1][1179] → 18(read: 10) r3[1][1213] → 12(read: 9) r3[1][1361] → 6(read: 7)

## Performance evaluation

In this section, we present the performance evaluation of the proposed algorithm. The performance of the proposed approach is compared against BASE [25] and DeClone [25].

### A. Experimental setup

We use simulation to analyze the performance of the proposed clone detection and determination approach. In the experiment, the data has been generated using Binomial distribution to illustrate the tag read count in an epoch as used in [48]. The performance of the proposed approach is compared against BASE [25] and DeClone [25] in terms of execution time and detection accuracy. The clone detection accuracy is measured via the ratio of collisions and total readings as in following Eqs 2 and 3:

$$Error\;ratio=\frac{Collisions}{Total\;readings}\times 100$$ (2)

Cloneaccuracy=100−Errorratio (3)

In this study, the collisions represent slot collisions that used to indicate probable clone due to similar hash digest value. Eq 4 is used to measure clone detection accuracy (*CDA*) for MCH.

$$CDA=\frac{Number\;of\;clones}{Number\;of\;collided\;slots}\times 100$$ (4)

Before executing the comparison, empirical work is done to the proposed approach for determining ideal bucket size in accordance to the appropriate packing density. Note that a slight modification is made to DeClone and BASE algorithms, however still based towards hash collision in Aloha-based approach due to existence of similar EPC. The modification is around simulation of the approaches in distributed environment as suggested.

### B. Ideal bucket size in accordance to packing density

As a rule of thumb, it is often found that collisions become unacceptably frequent if packing density exceeds 70% [50]. In other words, packing density is better to be lower than 70%. In the experiment, execution time of MCH is measured with a few sets of bucket size (*bs*) in 60% packing density. The following packing density Eq 5 as in [50] is used in this study to measure quantity of home bucket required for 60% packing density.

$$PD=\frac{M}{bs*K}\left(which\;must\;be\leq 1\right)$$ (5)

Assume that there are *K* home buckets, each has a capacity of *bs* records and *M* records are put into the file. Based on Eq 5, Table 7 shows the measurement of home bucket quantity for *M* records. Since we measure up to 10,000 numbers of readings for 60% packing density, 1667 home buckets were applied in each *CM* sketch. However, for any selected *bs* in conjunction with packing density (*PD*), there will be expected file overflow which is not discuss in this study.

**Table 7:** Home bucket quantity for bucket size = 10.

Bucket Size, bs = 10					
Number of records, M	% Packing Density				
	50	55	60	65	70
	Home Bucket Quantity, K				
1,000	200	182	167	154	143
2,000	400	364	333	308	286
3,000	600	545	500	462	429
4,000	800	727	667	615	571
5,000	1000	909	833	769	714
6,000	1200	1091	1000	923	857
7,000	1400	1273	1167	1077	1000
8,000	1600	1455	1333	1231	1143
9,000	1800	1636	1500	1385	1286
10,000	2000	1818	1667	1538	1429
:	:	:	:	:	:
20,000	4000	3636	3333	3077	2857

### C. Comparative analysis of execution time

**Fig 5:**
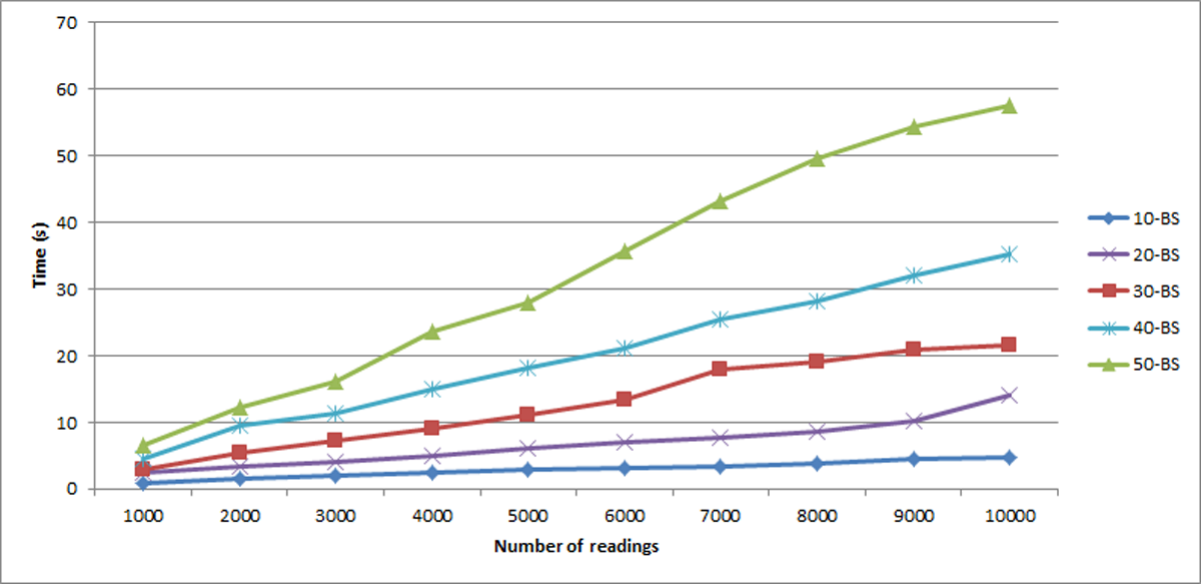
Execution time of MCH with 60% packing density and bucket sizes *bs* = 10, *bs* = 20, *bs* = 30, *bs* = 40 and *bs* = 50.

Extensive experiment is performed to find out *bs* that will return faster execution time. Fig 5 below shows the execution time of MCH using different number of bucket size *bs = 10*, *bs = 20*, *bs = 30*, *bs = 40* and *bs = 50*. The number of readings varied from 1,000 to 10,000 with increment of 1,000 for each sample. For all *bs* values, the result shows *bs* = 10 produced faster execution time with increment in the number of readings. Therefore, this measurement is used as the baseline in the next experiments to get the best results.

**Fig 6:**
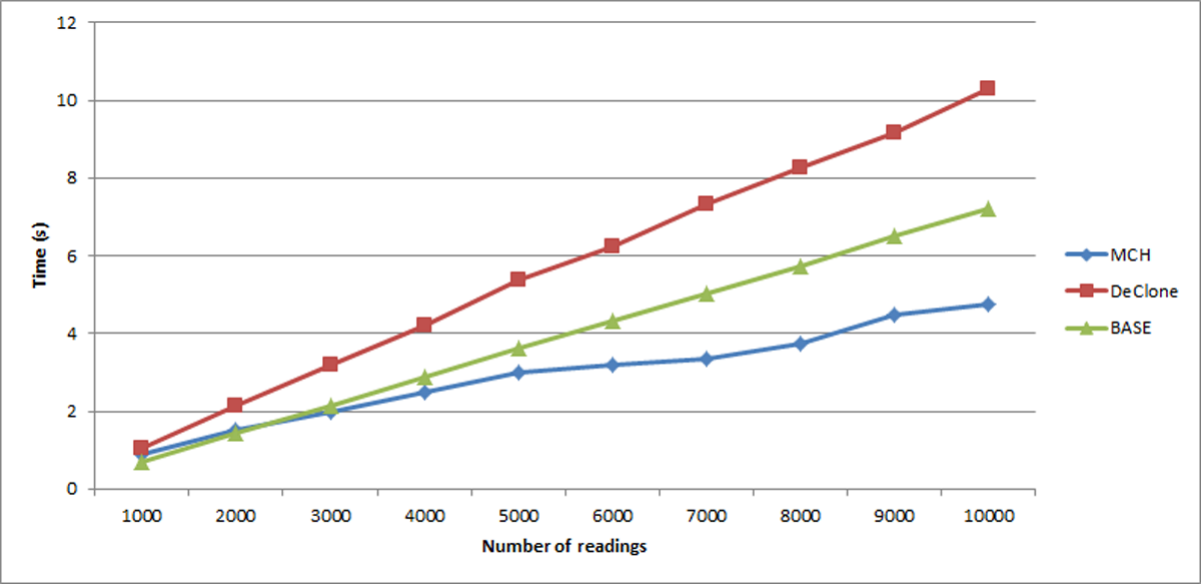
Comparison of execution times for detecting clone tag.

Fig 6 shows the execution time of the three approaches for the tag reading in the range of 1,000 to 10,000 readings. On average, all the approaches: MCH, BASE and DeClone took linear execution time with respect to the system scale. BASE and DeClone took longer time to detect the clone tag as compared to MCH. DeClone takes longer execution time because it has to perform the breadth first traversal for every single collision occurred before determining possibility of clone. Therefore, it involves a great amount of execution time for detecting clone tag.

### D. Clone detection accuracy

In this section, we study the clone detection accuracy of the BASE, DeClone and MCH under varying clone ratio.

**Fig 7:**
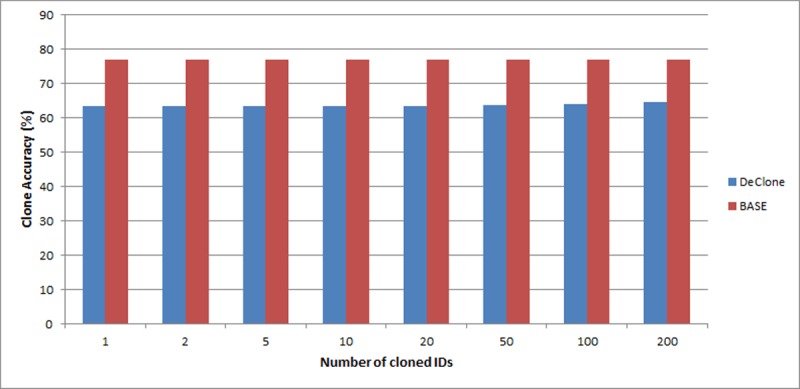
Comparison of clone detection accuracy between DeClone and BASE in varying number of clone IDs.

Fig 7 illustrates the performance of the BASE and DeClone under varying clone ratio in 10,000 readings. The number of clone EPCs varied from 1 to 200 which make up 2% of the readings. Fig 7 shows that as the number of cloned EPCs increases, the BASE algorithm tends to be more accurate in detecting clone tags than the DeClone approach. However, BASE is not able to find which EPCs are the clones since it just compare the sum of the tags in the system against the total EPC (clone attack makes the tag quantity to exceed the actual EPC quantity). Since the fluctuations rates are too small between the different numbers of clone EPCs, the changes in graph is not really obvious.

**Fig 8:**
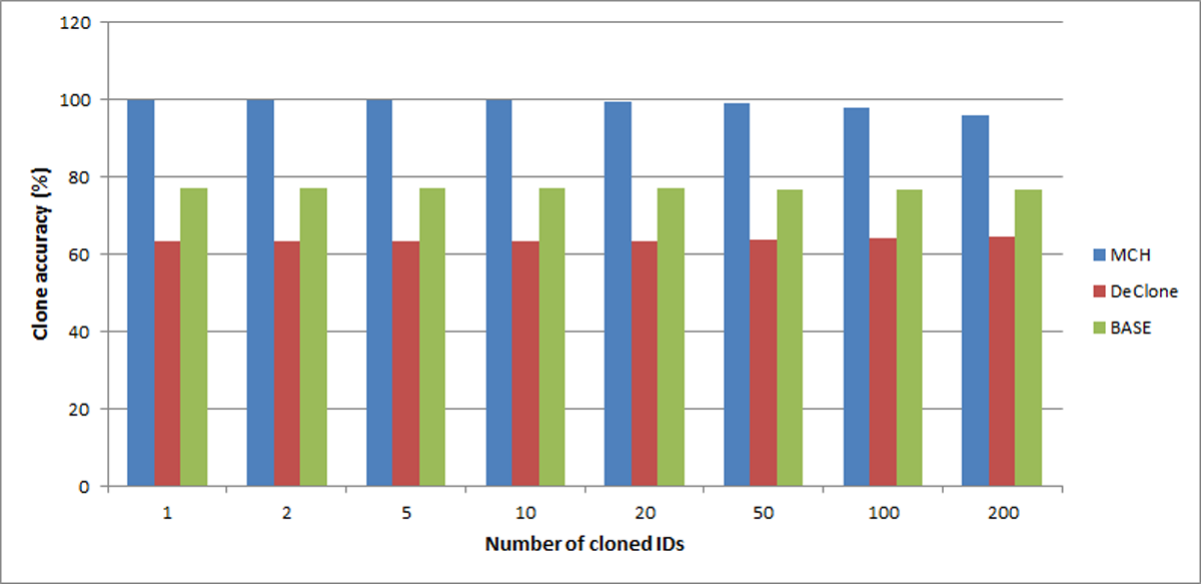
Comparison of clone detection accuracy between MCH, DeClone and BASE in varying number of clone IDs.

Fig 8 shows that MCH obtains higher accuracy for detecting the clone tag as compared to DeClone and BASE. Furthermore, MCH can precisely determine at which reader the clone tag exists (as discussed in example case study in section Clone Tag Detection Algorithm) under varying clone ratio in 10,000 readings. MCH outperforms the DeClone and BASE approaches in RFID tag clone detection accuracy as much as 99% in average while DeClone 64% and BASE 77%. Overall, detection accuracy of all approaches observed including MCH is getting reduced when number of clone increases. This is due to upturn of slots collision that indicate probable clone when the clone number growths. The slots collision is not yet determine the clone really exist because the hash function used can also produce slots collision when hashing different EPCs.

## Conclusion and future directions

In this paper, the problem of RFID clone tag detection has been studied and a new approach based on modified count-min sketch vector is proposed. Performance of the proposed approach is compared with the other related existing approaches. The results illustrate that the proposed approach performs faster than the baseline approaches in the experiments efficiency and better accuracy under varying clone ratio. The implementation of dual verification strategy (consistent hash collision and tag reading frequency) in the proposed approach produces as much as 99% in RFID tag clone detection accuracy than the other baseline approaches. For future work, this study plans to apply dynamic hashing together with the count-min sketch vector. This will help to accommodate the growth and shrinking of the file size over time. Even adding up in complexity, dynamic hashing advantages in minimizing space overhead since no slot need to be reserved for future use as implemented in static hashing. The proposed approach in this study is using 2D dynamic array and bucket with *d* hash functions. If bucket size exceeds the limit, another strategy will need to be used. Hashing with chaining is applied in the proposed approach and its theoretical advantage is it does not limit the bucket size. For improvement, the approach can exclude measurement on the bucket size to overcome limitation on bucket size. Without a pre-defined number of buckets not to exceed, the bucket will not overflow. A short linear search of the linked list is still needed, but if the hash function uniformly distributes the items, the list should not be very long. Presently, the algorithm is designed for controlled environment where there is time boundary for each tag to arrive at each location. For future improvement, the approach would consider an open environment for wider deployment.

## References

[1] J. Abawajy, “Enhancing RFID tag resistance against cloning attack,” in *Network and System Security*, 2009. NSS’09. *Third International Conference on*, 2009, pp. 18–23.

[2] Huang Y., Xu Y., Qi S., Fang X., and Yin X., “Recent Patents on RFID-Based Logistics Management Systems,” *Recent Patents Mech*. *Eng*., vol. 9, no. 1, pp. 26–36, 2016.

[3] Kamaludin H., Mahdin H., and Abawajy J. H., “Filtering Redundant Data from RFID Data Streams,” *J*. *Sensors*, vol. 2016, 2015. 10.3390/s111009863 PMC3231251 22163730

[4] Mahdin H., Kamaludin H., Saedudin R. D. R., Omar A. H., Kasim S., and Jailani J., “The Application of RFID System in Water Level Monitoring,” *Int*. *J*. *Adv*. *Sci*. *Eng*. *Inf*. *Technol*., vol. 7, no. 4–2, pp. 1522–1527, 2017.

[5] N. Alzahrani and N. Bulusu, “Securing Pharmaceutical and High-Value Products against Tag Reapplication Attacks Using NFC Tags,” in Smart Computing (SMARTCOMP), 2016 IEEE International Conference on, 2016, pp. 1–6.

[6] Tan X., Dong M., Wu C., Ota K., Wang J., and Engels D., “An Energy-Efficient ECC Processor of UHF RFID Tag for Banknote Anti-Counterfeiting,” *IEEE Access*, 2016.

[7] Ray B. R., Chowdhury M. U., and Abawajy J. H., “Secure Object Tracking Protocol for the Internet of Things,” *IEEE Internet Things J*., vol. 3, no. 4, pp. 544–553, 2016.

[8] Ray B. R., Abawajy J., Chowdhury M., and Alelaiwi A. A., “Universal and secure object ownership transfer protocol for the Internet of Things,” *Futur*. *Gener*. *Comput*. *Syst*., p., 2017.

[9] D. Zanetti, S. Capkun, and A. Juels, “Tailing RFID Tags for Clone Detection.,” in Network and Distributed System Security Symposium (NDSS), 2013.

[10] Huang J., Li X., Xing C.-C. C., Wang W., Hua K., and Guo S., “DTD: A Novel Double-Track Approach to Clone Detection for RFID-enabled Supply Chains,” *IEEE Trans*. *Emerg*. *Top*. *Comput*., vol. 6750, no. c, pp. 1–1, 2015.

[11] L. Mirowski, “Exposing clone RFID tags at the reader,” in Trust, Security and Privacy in Computing and Communications (TrustCom), 2013 12th IEEE International Conference on, 2013, pp. 1669–1674.

[12] Ronald Quirk, “E-Pedigree’s Evolution—2007-03-05—Page 1—RFID Journal,” RFID Journal, 2007. [Online]. Available: http://www.rfidjournal.com/articles/view?3109. [Accessed: 20-Apr-2017].

[13] Farash M. S., Nawaz O., Mahmood K., Chaudhry S. A., and Khan M. K., “A Provably Secure RFID Authentication Protocol Based on Elliptic Curve for Healthcare Environments,” *J*. *Med*. *Syst*., vol. 40, no. 7, p. 165, 5 2016 doi: 10.1007/s10916-016-0521-6 27221283 10.1007/s10916-016-0521-6

[14] Chen J., Miyaj A., Sato H., and Su C., “Improved Lightweight Pseudo-Random Number Generators for the Low-Cost RFID Tags,” in *Trustcom/BigDataSE/ISPA*, *2015 IEEE*, 2015, vol. 1, pp. 17–24.

[15] Halamka J., Juels A., Stubblefield A., and Westhues J., “The security implications of VeriChip cloning,” *J*. *Am*. *Med*. *Informatics Assoc*., vol. 13, no. 6, pp. 601–607, 2006. 10.1197/jamia.M2143 PMC1656959 16929037

[16] Bono S., Green M., Stubblefield A., Juels A., Rubin A. D., and Szydlo M., “Security Analysis of a Cryptographically-Enabled RFID Device.,” in *Usenix Security*, 2005, vol. 5, pp. 1–16.

[17] A. Ilic, M. Lehtonen, F. Michahelles, and E. Fleisch, “Synchronized Secrets Approach for RFID-enabled Anti-Counterfeiting,” in Demo at Internet of Things Conference, 2008.

[18] M. Lehtonen, D. Ostojic, A. Ilic, and F. Michahelles, “Securing RFID systems by detecting tag cloning,” in International Conference on Pervasive Computing, 2009, pp. 291–308.

[19] K. Bu, X. Liu, and B. Xiao, “Fast cloned-tag identification protocols for large-scale RFID systems,” in Quality of Service (IWQoS), 2012 IEEE 20th International Workshop on, 2012, pp. 1–4.

[20] Choi S. H., Yang B., Cheung H. H., and Yang Y. X., “RFID tag data processing in manufacturing for track-and-trace anti-counterfeiting,” *Comput*. *Ind*., vol. 68, pp. 148–161, 2015.

[21] Mahdin H. and Abawajy J., “An Approach for Removing Redundant Data from RFID Data Streams,” *Sensors*, vol. 11, no. 10, pp. 9863–9877, 1 2011 doi: 10.3390/s111009863 22163730 10.3390/s111009863 PMC3231251

[22] Yimin G., Shundong L., Jiawei D., and Sufang Z., “Deterministic cloned tag detection protocol for anonymous radio-frequency identification systems,” *Inf*. *Secur*. *IET*, vol. 10, no. 1, pp. 28–32, 2016.

[23] EPCglobal, “Pedigree Ratified Standard,” 2007. [Online]. Available: http://www.gs1.org/sites/default/files/docs/epc/pedigree_1_0-standard-20070105.pdf. [Accessed: 05-Sep-2016].

[24] Choi S. H., Cheung H. H., Yang B., and Yang Y. X., “Item-level RFID for retail business improvement,” in *RFID Technology Integration for Business Performance Improvement*, IGI Global, Hershey, Pennsylvania, USA, 2014, pp. 1–26.

[25] Bu K., Xu M., Liu X., Luo J., Zhang S., and Weng M., “Deterministic Detection of Cloning Attacks for Anonymous RFID Systems,” *IEEE Trans*. *Ind*. *Informatics*, vol. 11, no. 6, pp. 1–1, 2015.

[26] EPCglobal, “EPCglobal | GS1,” 2017. [Online]. Available: https://www.gs1.org/epcglobal. [Accessed: 05-Oct-2017].

[27] EPCglobal, “Application Level Events (ALE) Standard | GS1,” 2017. [Online]. Available: https://www.gs1.org/ale. [Accessed: 05-Oct-2017].

[28] Sweeney P. J., *RFID for Dummies*. John Wiley & Sons, 2010.

[29] Fabrlzlo Pllato, “Nokia 5140 RFID Reader | Mobile Magazine,” 2004. [Online]. Available: https://www.mobilemag.com/2004/03/16/nokia-5140-rfid-reader/. [Accessed: 07-Mar-2017].

[30] Michael Kwan, “Nokia 6131 With RFID For Tap-And-Go Payment | Mobile Magazine,” 2007. [Online]. Available: https://www.mobilemag.com/2007/11/23/nokia-6131-with-rfid-for-tap-and-go-payment/. [Accessed: 07-Mar-2017].

[31] Frequentz Information Center, “ePedigree Overview,” 2014. [Online]. Available: http://frequentz.com/infocenter/iris/3.2/ePedigree_Overview.htm. [Accessed: 20-Apr-2017].

[32] A. Juels, “Strengthening EPC tags against cloning,” in Proceedings of the 4th ACM workshop on Wireless security, 2005, pp. 67–76.

[33] Abawajy J. and Fernando H., “Policy-based SQLIA detection and prevention approach for RFID systems,” *Comput*. *Stand*. *Interfaces*, vol. 38, pp. 64–71, 2015.

[34] Abawajy J., “SQLIA detection and prevention approach for RFID systems,” *J*. *Syst*. *Softw*., vol. 86, no. 3, pp. 751–758, 2013.

[35] Ray B. R., Abawajy J., and Chowdhury M., “Scalable RFID security framework and protocol supporting Internet of Things,” *Comput*. *Networks*, vol. 67, pp. 89–103, 2014.

[36] M. Lehtonen, F. Michahelles, and E. Fleisch, “How to detect cloned tags in a reliable way from incomplete RFID traces,” in RFID, 2009 IEEE International Conference on, 2009, pp. 257–264.

[37] Aggarwal C. C., Ashish N., and Sheth A., “The Internet of Things: A Survey from the Data-Centric Perspective,” in *Managing and Mining Sensor Data*, Aggarwal C. C, Ed. Boston, MA: Springer US, 2013, pp. 383–428.

[38] Liu X. et al., “RFID Estimation with Blocker Tags,” *IEEE/ACM Trans*. *Netw*., 2016.

[39] Bu K., Liu X., Luo J., Xiao B., and Wei G., “Unreconciled collisions uncover cloning attacks in anonymous RFID systems,” *Inf*. *Forensics Secur*. *IEEE Trans*., vol. 8, no. 3, pp. 429–439, 2013.

[40] Huang J., Li X., Xing C.-C., Wang W., Hua K., and Guo S., “DTD: A novel double-track approach to clone detection for RFID-enabled supply chains,” *IEEE Trans*. *Emerg*. *Top*. *Comput*., vol. 5, no. 1, pp. 134–140, 2017.

[41] A. Goyal, J. Jagarlamudi, H. Daumé III, and S. Venkatasubramanian, “Sketching techniques for large scale NLP,” in Proceedings of the NAACL HLT 2010 Sixth Web as Corpus Workshop, 2010, pp. 17–25.

[42] Cormode G. and Muthukrishnan S., “An improved data stream summary: the count-min sketch and its applications,” *J*. *Algorithms*, vol. 55, no. 1, pp. 58–75, 2005.

[43] Chen J. and Chen P., “Sequential Pattern Mining for Uncertain Data Streams using Sequential Sketch,” *J*. *Networks*, vol. 9, no. 2, pp. 252–258, 2014.

[44] Kaneiwa K. and Kudo Y., “A sequential pattern mining algorithm using rough set theory,” *Int*. *J*. *Approx*. *Reason*., vol. 52, no. 6, pp. 881–893, 2011.

[45] X. Zhang, C. Lan, and A. Perrig, “Secure and Scalable Fault Localization under Dynamic Traffic Patterns,” in Security and Privacy (SP), 2012 IEEE Symposium on, 2012, pp. 317–331.

[46] F. Rusu and A. Dobra, “Statistical analysis of sketch estimators,” in Proceedings of the 2007 ACM SIGMOD international conference on Management of data, 2007, pp. 187–198.

[47] Mark Roberti, “How Many Tags Can Be Read By an RFID Reader at One Time?,” RFID Journal, 2011. [Online]. Available: http://www.rfidjournal.com/blogs/experts/entry?8958. [Accessed: 29-Jun-2016].

[48] S. R. Jeffery, U. C. Berkeley, M. J. Franklin, M. Garofalakis, and M. J. Franklin, “Adaptive cleaning for RFID data streams,” in Proceedings of the 32nd international conference on Very large data bases, 2006, pp. 163–174.

[49] N. Rivetti, Y. Busnel, and A. Mostéfaoui, “Efficiently summarizing data streams over sliding windows,” in *Network Computing and Applications (NCA), 2015 IEEE 14th International Symposium on*, 2015, pp. 151–158.

[50] “Hashing and Overflow Strategies.” [Online]. Available: http://pluto.ksi.edu/~cyh/cis501/ch05b.htm. [Accessed: 15-Jul-2016].

